# Chronic urticaria: a focus on pathogenesis

**DOI:** 10.12688/f1000research.11546.1

**Published:** 2017-07-11

**Authors:** Riccardo Asero, Alberto Tedeschi, Angelo Valerio Marzano, Massimo Cugno

**Affiliations:** 1 Ambulatorio di Allergologia, Clinica San Carlo, Paderno Dugnano (Milano), Italy; 2 Ambulatorio di Allergologia e Immunologia Clinica, Ospedale Fatebenefratelli e Oftalmico, Milano, Italy; 3 Unità di Dermatologia, Dipartimento di Fisiopatologia Medico Chirurgica e dei Trapianti, Università degli Studi di Milano, Fondazione IRCCS Ca’ Granda Ospedale Maggiore Policlinico, Milano, Italy; 4 Medicina Interna, Dipartimento di Fisiopatologia Medico Chirurgica e dei Trapianti, Università degli Studi di Milano, Fondazione IRCCS Ca’ Granda Ospedale Maggiore Policlinico, Milano, Italy

**Keywords:** Chronic Uritcaria, wheals, autologous serum skin test, Omalizumab

## Abstract

Chronic urticaria is a spontaneous or inducible group of diseases characterized by the occurrence of wheals (and, in about half of cases, angioedema) for more than 6 weeks. These are rather frequent conditions that may severely affect patients’ quality of life and sometimes represent a challenge for doctors as well. The causes of chronic urticaria are still poorly defined, although there is growing evidence that different biologic systems including immunity, inflammation, and coagulation may take part in the pathomechanism eventually leading to mast cell and basophil degranulation and hence to wheal formation. This review will discuss the main findings that are (slowly) shedding light on the pathogenesis of this disorder.

## Introduction

Chronic urticaria, defined as the recurrent occurrence of wheals with or without angioedema for longer than 6 weeks, can be classified into spontaneous or inducible based on the absence or presence of identifiable physical stimuli able to elicit the skin lesions ^1^. While there is little doubt that the release of histamine by mast cells and basophils represents the final stage in the pathomechanisms involved in this group of diseases, there is still some uncertainty about the factors activating these cells and eventually inducing their degranulation. Several lines of evidence indicate that different biologic systems like immunity, inflammation, and coagulation may contribute to a common mechanism leading to wheal generation. This short commentary will summarize the current knowledge about the mechanisms theoretically leading to histamine release in chronic urticaria.

## Established facts

The bulk of studies carried out during the last 30 years have shown that different biologic systems (i.e. autoimmunity, auto-allergy, inflammation, and coagulation) may concur to produce the wheal and flare lesions as well as the angioedema that characterize chronic urticaria. For clarity, these biologic systems, which are largely linked with each other, along with the main cell lines involved in either their activation or their behavior as effector cells will be considered singularly in the following subsections.

### Autoreactivity/autoimmunity

More than 30 years ago, Grattan and co-workers demonstrated that the intradermal injection of autologous serum induces a wheal-and-flare reaction in a large proportion of patients with chronic urticaria ^2^; this led them to conclude that chronic urticaria is characterized by circulating histamine-releasing factors. Since then, a positive autologous serum skin test (ASST) has been considered as a marker of “autoreactivity”. This *in vivo* test is quite easy to perform and seems sufficiently specific for chronic urticaria, as normal subjects do not show any skin reactivity ^3^, although it may score positive in inducible urticarias such as cold urticaria ^4^ as well as in some other “allied” conditions, such as the multiple drug allergy syndrome and multiple hypersensitivity to non-steroidal anti-inflammatory drugs ^5, 6^. However, the ASST scores positive in only about one half of chronic urticaria patients ^7, 8^, leaving a large proportion of patients who do not show any autoreactivity despite their maybe severe and ongoing disease.

Following the first observation on skin reactivity to autologous serum that remains a cornerstone in urticaria research, the second important step in the definition of the pathogenic mechanism was the demonstration of histamine-releasing autoantibodies about 20 years ago, suggesting an autoimmune origin. This was based on the detection of circulating and functionally active IgG autoantibodies directed against either the high-affinity IgE receptor (FcεRI) present on both mast cells and basophils (in most cases) or membrane-bound IgE (in a minority of patients) ^9– 12^.

Although this was a fascinating explanation for the ongoing histamine release, several subsequent studies found that this mechanism is in fact viable in only a minority of patients ^8, 13^. Furthermore, both types of autoantibodies have been detected with similar frequency in chronic urticaria patients and controls without chronic urticaria ^13^. Nonetheless, in more recent times, the autoimmune pathogenesis hypothesis has received new, indirect but strong support from the detection of circulating autoreactive CD4 ^+^ T cells that proliferate in response to FcεRI in >50% of patients with chronic urticaria examined ^14^.

### Basophils: their pathogenic role and use in chronic urticaria diagnosis

Although there is little doubt that the mast cell is the main final effector cell in chronic urticaria, basophils and their role in the disease have received much attention during the last decade. It is well known that active chronic urticaria is characterized by basopenia due to the elective recruitment of circulating basophils into the skin lesions ^15^ and that basopenia is directly related to disease severity. Recent studies found that chronic urticaria blood basophils show a reduced surface expression of CRTH2 (the chemoattractant receptor–homologous molecule expressed on TH2 cells), the receptor for prostaglandin D2 (PGD2). It is therefore possible that decreased CRTH2 levels on basophils in patients with chronic urticaria are due to *in vivo* PGD2-mediated activation, suggesting the engagement of CRTH2 in patients with chronic urticaria ^16^. Furthermore, basophils from chronic urticaria patients show different phenotypes (either responder or non-responder, based on the degranulation response to polyclonal goat anti-human IgE) that seem independent of the presence of IgG autoantibodies to IgE or FcεRI and seem stable during active disease ^13, 17^, and their IgE receptor-mediated degranulation is enhanced during disease remission ^13, 17^.

The finding that at least a proportion of chronic urticaria patients show autoimmune phenomena targeting mast cells and basophils has led to the investigation of the diagnostic accuracy of basophil-based *in vitro* tests aiming to diagnose autoimmunity. The first test used was the time-consuming and cumbersome basophil histamine release assay (BHRA), first introduced in the 1960s and subsequently marketed as a more convenient testing kit ^18^; on the other hand, the observation that chronic urticaria sera were able to induce the surface expression of basophil activation markers such as CD63 ^19^ led to the investigation of the diagnostic usefulness of the basophil activation test (BAT) measuring CD63 and CD203c surface expression by flow cytometry ^20, 21^. However, in view of the fact that no more that 50% of chronic urticaria patients have an autoimmune disease and of the intrinsic variability of basophil releasability, the clinical usefulness of these tests has been questioned ^22^ and they have not reached the status of routine investigation for this disease.

### Auto-allergy

Another important step was the discovery of specific IgE for thyroid peroxidase (TPO) for dsDNA and ssDNA, which has recently provided a different mechanism potentially able to promote the survival, proliferation, and activation of mast cells ^23^. This pathogenic mechanism could be classified as “auto-allergic” and autoimmune at the same time. However, the proportion of patients in whom such IgE autoantibodies can be detected is limited, and TPO-specific IgE autoantibodies are generally associated with the more common TPO IgG autoantibodies that characterize patients with Hashimoto’s thyroiditis which are found in a minority of patients. However, research in this field is very active, and it cannot be excluded that other “auto-allergens” will be detected in larger proportions of patients with chronic urticaria in the near future ^24^.

### Inflammation

It is increasingly clear that chronic urticaria is characterized by a systemic pro-inflammatory state. The studies showing increased levels of different single markers of inflammation (e.g. matrix metalloproteinase and others) that have appeared in the medical literature over the years will not be reviewed in detail here. Recent studies showed the co-existence of chronic urticaria and metabolic syndrome (MS) in a Korean population ^25^; patients with both chronic urticaria and MS were older, had a more severe disease, had higher levels of inflammation markers (i.e. eosinophil cationic protein [ECP], tumor necrosis factor [TNF]-alpha, and complement), and, interestingly, more frequently scored negative on the ASST than those without MS. In another Asian-based population study, chronic urticaria was more frequent among subjects with a prior diagnosis of hyperlipidemia ^26^. Recently, chronic urticaria patients were found to show increased levels of a series of adipokines (lipocalin-2, TNF-alpha, IL-6, and IL-10) but lower levels of adiponectin ^27^. The findings with IL-6 are in keeping with a previous study ^28^ showing an association between IL-6 and disease severity.

### Coagulation

The observation that the autologous plasma skin test (APST) may score positive in some ASST-negative patients ^29^ has prompted investigation of the coagulation system in patients with chronic urticaria. Specific studies have shown that the coagulation cascade is activated in chronic urticaria and involves the extrinsic pathway first and the intrinsic pathway second ^29– 32^. The process seems to be triggered by the hyper-expression of tissue factor by activated eosinophils ^33^ and parallels disease activity. IgG autoantibodies to the low-affinity IgE receptor FcεRII, which is present on the membrane of eosinophils, have been detected in quite a large proportion of patients (about 65%) with chronic urticaria ^34^ and might be involved in the activation of such cells with subsequent release of major basic protein and other mediators causing mast cell degranulation. The activation of the coagulation cascade might potentially play a relevant pathogenic role if one considers that thrombin can markedly increase the vascular permeability and is a potent inducer of mast cell degranulation in experimental models. The activation of the coagulation cascade occurs in other skin disorders characterized by an increase of vascular permeability, such as angioedema due to C1-inhibitor deficiency and bullous pemphigoid ^35, 36^, but also in a wide spectrum of systemic diseases, such as disseminated intravascular coagulation ^37^, deep venous thrombosis ^38^, and endotoxemia ^39^, all prothrombotic conditions that are not characterized by urticaria or edema. However, the activation of coagulation should not necessarily be considered a non-specific phenomenon but rather a common intermediate step occurring in the pathophysiology of different diseases. Thus, the involvement of coagulation is another piece of truth unable to explain the whole story.

### Other mechanisms

Quite recently, we were able to show that sera from patients with chronic urticaria induce significant activation of mast cells lacking the high-affinity IgE receptor, irrespective of the autoreactivity status or of the presence/absence of circulating autoantibodies ^40^. This fact suggests a pathomechanism that bypasses the IgE receptor mechanism. In subsequent studies, we showed that low-molecular-weight circulating factors (molecular weight about 30 kDa) may be involved in the activation of mast cells in chronic urticaria patients ^41^, thus confirming very old data by Grattan and co-workers ^42^.

## Using the response to omalizumab as a means to distinguish different chronic urticaria phenotypes

Omalizumab was recently licensed for the treatment of chronic urticaria. All three phase III studies as well as the real-life studies carried out in large numbers of patients have demonstrated that this humanized monoclonal IgG antibody to IgE is highly effective in a large proportion of patients with chronic urticaria who don’t show any clinical response to second-generation antihistamines given at doses even higher than licensed ones. The pattern of response to this drug has indirectly led to the categorization of patients with severe disease into “fast responders” (subjects who show a complete response as short as 3–7 days after the first administration of the drug; these represent about 60–70% of patients), “slow responders” (subjects who need three to five monthly administrations before showing a significant clinical response; about 10–20% of cases), and “non-responders” (about 10–20% of cases) ^43^. It is easy to speculate that different pathogenic mechanisms might underlie the different patterns of response. Several potential markers of response to omalizumab treatment have been investigated so far. In one study looking at the potential use of a CD203c assay (the so-called BAT) as a biomarker of responsiveness to omalizumab, it was found that the lack of basophil CD203 upregulating activity was associated with a higher likelihood of response to the drug ^44^. This observation seems to agree with the results of another study finding that a positive response to omalizumab was associated with a negative histamine release assay ^45^. On the other hand, Gericke and co-workers found that a positive BHRA, which was associated with a positive ASST, was frequently observed in “slow responders” to omalizumab ^43^. Another interesting observation is that omalizumab has been found to be effective not only in the spontaneous form but also in several inducible forms of chronic urticaria ^46, 47^. This suggests that the drug interferes with a common pathogenic mechanism underlying a proportion of both spontaneous and inducible urticarias. Notably, the fact that the symptomatic cold urticaria, a frequent chronic inducible urticaria, can be passively transmitted to normal subjects via the serum of affected individuals has been known for many years. Since the mechanism of action of omalizumab in urticaria is all but elucidated, the next few years will probably shed some light on these questions.

## Conclusions

The pathogenesis of chronic urticaria is probably characterized by a multiplicity of mechanisms, including autoimmunity, auto-allergy, and coagulation, each of which may carry different weight in each patient. Much research is in progress, and the overall perception is that the solution to the puzzle is not too far away.
